# Severe Retinopathy of Prematurity Associated With Neurodevelopmental Disorder in Children

**DOI:** 10.3389/fped.2022.816409

**Published:** 2022-02-09

**Authors:** Young-Jin Choi, Eun Hee Hong, Yong Un Shin, Gi Hwan Bae, Inah Kim, Heeyoon Cho

**Affiliations:** ^1^Department of Pediatrics, Hanyang University Guri Hospital, Guri, South Korea; ^2^Department of Ophthalmology, Hanyang University Guri Hospital, Guri, South Korea; ^3^Department of Ophthalmology, Hanyang University College of Medicine, Seoul, South Korea; ^4^Department of Occupational and Environment Medicine, Hanyang University College of Medicine, Seoul, South Korea

**Keywords:** premature infants, retinopathy of prematurity, neurodevelopmental disorders, developmental delay, gestational age

## Abstract

**Objective:**

This study aimed to investigate whether severe retinopathy of prematurity (ROP) could be an association factor for neurodevelopmental disorders in premature infants without other risk factors—such as congenital anomalies, birth injuries, and neurological diseases—that may cause developmental delay.

**Methods:**

We used health claims data recorded between 2007 and 2018 in the Korean National Health Insurance Service (KNHIS) database. We recruited a total of 18,256 premature infant born between 2007 and 2008 without congenital anomaly or birth injury (with ROP 6,995, without ROP 11,261) and divided them into four groups as follows: Group A, 209 extremely premature infants [gestational age (GA) < 28] with mild ROP; Group B, 75 extremely premature infants (GA < 28) with severe ROP; Group C, 6,510 other premature infants (28 ≤ GA <37)with mild ROP; and Group D, 201 other premature infants (28 ≤ GA < 37) with severe ROP. Using regression analysis, we analyzed whether there was a correlation between ROP prevalence, severity, and developmental delay in premature infants without other risk factors.

**Results:**

The prevalence of developmental delay, according to GA and ROP severity, was higher in patients with severe ROP than in the other patients. The prevalence gradually decreased after birth. Among extremely premature infants with ROP, those with severe ROP had a 3.082-fold higher association with neurodevelopmental complications than those with mild ROP (*p* < 0.001). Compared with other premature infants with ROP, those with severe ROP had a 3.269-fold higher association with neurodevelopmental complications than those with mild ROP.

**Conclusion:**

The severity of ROP may be associated with neurodevelopmental disorders in premature infants.

## Introduction

Recently, the birth rate of premature infants has been steadily increasing in South Korea; however, owing to the medical development of the neonatal intensive care unit (NICU), their survival has also increased (1–4). Since premature infants are more likely to have neurodevelopmental disorders than full-term infants and infants with a normal birth weight, the long-term neurodevelopmental prognosis of premature infants has gained attention (4–6). Early detection of neurodevelopmental delay in premature infants is important because early diagnosis and appropriate treatment can improve the patients' condition or alleviate their symptoms (4, 7). Therefore, the early identification of risk factors by conducting intensive developmental screening tests is imperative to classify high-risk groups for developmental delay (6, 8, 9).

Retinopathy of prematurity (ROP) is a disease wherein abnormal vascular proliferation occurs during retinal development in premature newborns (3, 10–12). As the incidence of premature births and low birth weight in newborns increases, the overall risk of ROP also increases (13, 14). Severe ROP is a major cause of childhood blindness, myopia, high myopia, disparity, amblyopia, and astigmatism strabismus. Additionally, continuous progression of ROP can lead to complications, such as secondary glaucoma and blindness, caused by retinal detachment secondary to fibrous tissue retinal traction. Nonetheless, most ROP cases undergo spontaneous regression during follow-up, without any special treatment (15). Recent studies have focused on the correlation between extraocular and neurodevelopmental complications, while previous studies have investigated the developmental and maturity status of the brain through imaging screening at a point in time (13, 16–18).

The age at diagnosis is different for each area of developmental delay in children; motor dysfunction is often detected early, while learning disabilities are only detected at an average age of 5–6 years (19). Thus, neurodevelopmental disorders should be assessed through continuous, long-term follow-up (7, 9), and are difficult to study using hospital-based data.

In this study, we aimed to investigate the association between ROP severity and neurodevelopmental disorders in children using the Korean National Health Insurance Service (KNHIS) database.

## Materials and Methods

This study was approved by the Institutional Review Board (IRB no. 2018-04-001) of Hanyang University Guri Hospital, Gyeonggi-do, South Korea. The requirement for written informed consent was waived due to the retrospective study design. The research was conducted according to the tenets of the Declaration of Helsinki.

We used health claims data recorded between 2007 and 2018 in the KNHIS database. In South Korea, the health security system provides healthcare coverage to all citizens. The KNHIS database covers all citizens in South Korea and includes data regarding diagnoses, procedures, prescription records, medical treatment records, sociodemographic characteristics, and direct medical costs for claims made (11).

### Participant Recruitment

We analyzed the health claims data recorded between 2007 and 2018 in the KNHIS database. In 2007, the National Health Screening Program for Infant and Children was initiated in Korea. Therefore, most children born after 2007 were regularly evaluated for development through health screening program (20). Consequently, we recruited premature infants born between 2007 and 2008 and followed them up for 10 years. Cases were identified according to the International Classification of Diseases, 10^th^ edition (ICD-10). The KNHIS database manages claims using the Korean Classification of Disease, 6^th^ edition, a modified version of the ICD-10 adapted for the Korean healthcare system (21).

A lower gestational age (GA) is associated with a higher probability of complications (22). Numerous other factors can cause neurological complications, such as birth injury and asphyxia. To account and adjust for possible errors, premature infants were divided into two groups: extremely premature infants (GA < 28 weeks, diagnostic code P07.2), with a high possibility of neurodevelopmental complications, and “other premature infants” (GA, 28–37 weeks, diagnostic code P07.3), with a lower possibility of developmental complications. A comparative analysis was then performed within groups with similar possibility of neurodevelopmental complications.

In this study, we try to analyze the hazard ratio (HR) of having neurodevelopmental problems according to the severity of ROP among patients who less likely to have neurological disorders.

Therefore, we excluded other conditions that can cause neurological complications. The following diagnostic codes were excluded:

- Q000–Q002: anencephaly
- Q010, Q011, Q012, Q018: encephalocele
- Q02: microcephaly
- Q03, Q031, Q038, Q039: hydrocephalus
- Q040–Q049: malformations of the brain
- P910–P919: cerebral ischemia, periventricular leukomalacia (PVL), coma, acquired hydrocephalus
- P941, P942, P948, P949: hypertonia, hypotonia, floppy baby, muscle tone disorder
- P100–P159: birth injury
- P520–P529: intraventricular hemorrhage (IVH), subependymal hemorrhage
- P200–P219: asphyxia.

### ROP

Retinopathy of prematurity was defined based on the diagnostic code (H35.1) within 180 days of the diagnosis of premature infants.

#### Severity of ROP

To classify ROP severity, patients were divided into two groups: mild and severe ROP. We defined patients who were diagnosed with ROP, but spontaneously healed without ophthalmic treatment, as the “mild ROP group.” By contrast, patients who underwent ROP treatment after diagnosis were defined as the “severe ROP group.”

Patients who underwent treatment were identified using procedure codes for pars plana vitrectomy (S5121-2), retinal detachment surgery (S5130), retinal photocoagulation (S5160), and cryopexy (S5140). We only included cases with the aforementioned procedure codes diagnosed within a year from ROP diagnosis to exclude treatment for other diseases. We assumed that the indications for treatment had been based on the treatment guidelines reported by the Early Treatment for Retinopathy of Prematurity Cooperative Group (ETROP) in 2003 (23): Indications for ROP treatment are type 1 ROPs (zone 1, any stage ROP with plus disease; zone 1, stage 3 ROP without plus disease; zone 2, stage 2 or 3 ROP with plus disease), and the treatment modalities include laser photocoagulation, cryotherapy, vitrectomy, or scleral bucking. Although there has been widespread use of intravitreal anti-vascular endothelial growth factor (VEGF) injections worldwide since the 2000–2010s (24, 25), the use of anti-VEGF in clinics began to be reported from the late 2000s in South Korea (26–29). And it was off-label use in South Korea, therefore, so it was not covered by the NHI service during the study period. Therefore, the treatment for ROP in the current study included the conventional treatment (laser photocoagulation, cryotherapy, vitrectomy, or scleral bucking).

**Figure 1:**
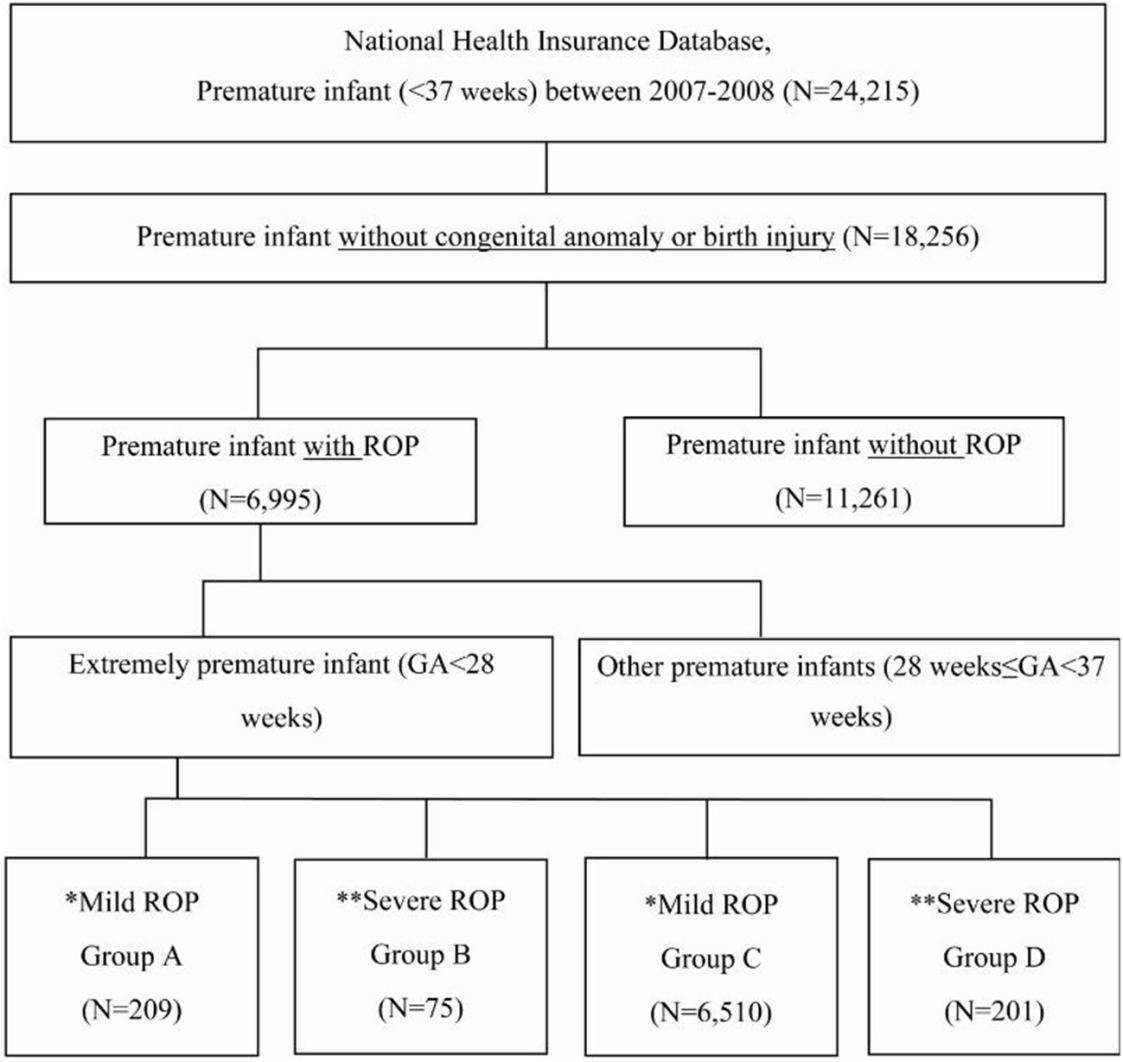
Enrollment and grouping of study patients according to gestational age and retinopathy of prematurity severity. GA, gestational age. *Mild ROP: spontaneously healed without special treatment. **Severe ROP: underwent treatment for ROP.

We divided the infants into four groups (Figure 1): Group A, extremely premature infants with mild ROP; Group B, extremely premature infants with severe ROP; Group C, other premature infants with mild ROP; and Group D, other premature infants with severe ROP.

### Neurodevelopmental Disorders

Neurodevelopmental disorders include three subcategories: neurocognitive function, speech and language, and motor function developmental disorders. Developmental disorders of neurocognitive function included delayed normal physiological development (R62.0), intellectual disability (F70.0–73.0, 78.0, 79.0), pervasive developmental disorder (F84.8, 84.9), and psychological developmental disorder (F89). Moreover, developmental disorders of speech and language included developmental disorders of speech and language (F80.0, 80.1, 80.8, 80.9), developmental disorders of scholastic skills (F81.9), and attention deficit (F90.0). Finally, motor developmental disorders included lack of coordination (R27.0, 27.8) and developmental disorders of motor function (F82, 83).

### Statistical Analyses

We collected data of patients diagnosed with ROP among premature infants born between 2007 and 2008 and then followed-up their health insurance data for 10 years to confirm the diagnosis of neurodevelopmental disorders. The annual period prevalence of neurodevelopmental disorders over 10 years was calculated. We investigated whether there was a difference in the prevalence between the severe ROP and mild ROP groups. Using regression analysis, we analyzed if there was an association between ROP prevalence and severity and developmental delay in premature infants without other risk factors. The annual period prevalence at each age was calculated by dividing the number of prevalent cases by population. A Cox proportional hazards regression model was used to calculate the HRs and 95% confidence intervals (CIs); HRs with CIs were estimated using logistic regression analysis adjusted for sex (male vs. female), year of diagnosis (2007 vs. 2008), income level (grouped based on income quintiles), and area of residence (metropolitan cities vs. others). The model included sex (male vs. female), income level of the patient's household (insurance payment classes: low vs. middle, high), area of residence (metropolitan cities vs. others), and year of premature diagnosis (2007 vs. 2008). Statistical significance was set at *p* < 0.05, and all analyses were performed using SAS version 9.4 (SAS Inc., Cary, NC, USA).

## Results

### Neurodevelopmental Disorders

#### Annual Prevalence of Total Neurodevelopmental Disorders After Birth Throughout the 10-Year Follow-Up Period

**Figure 2:**
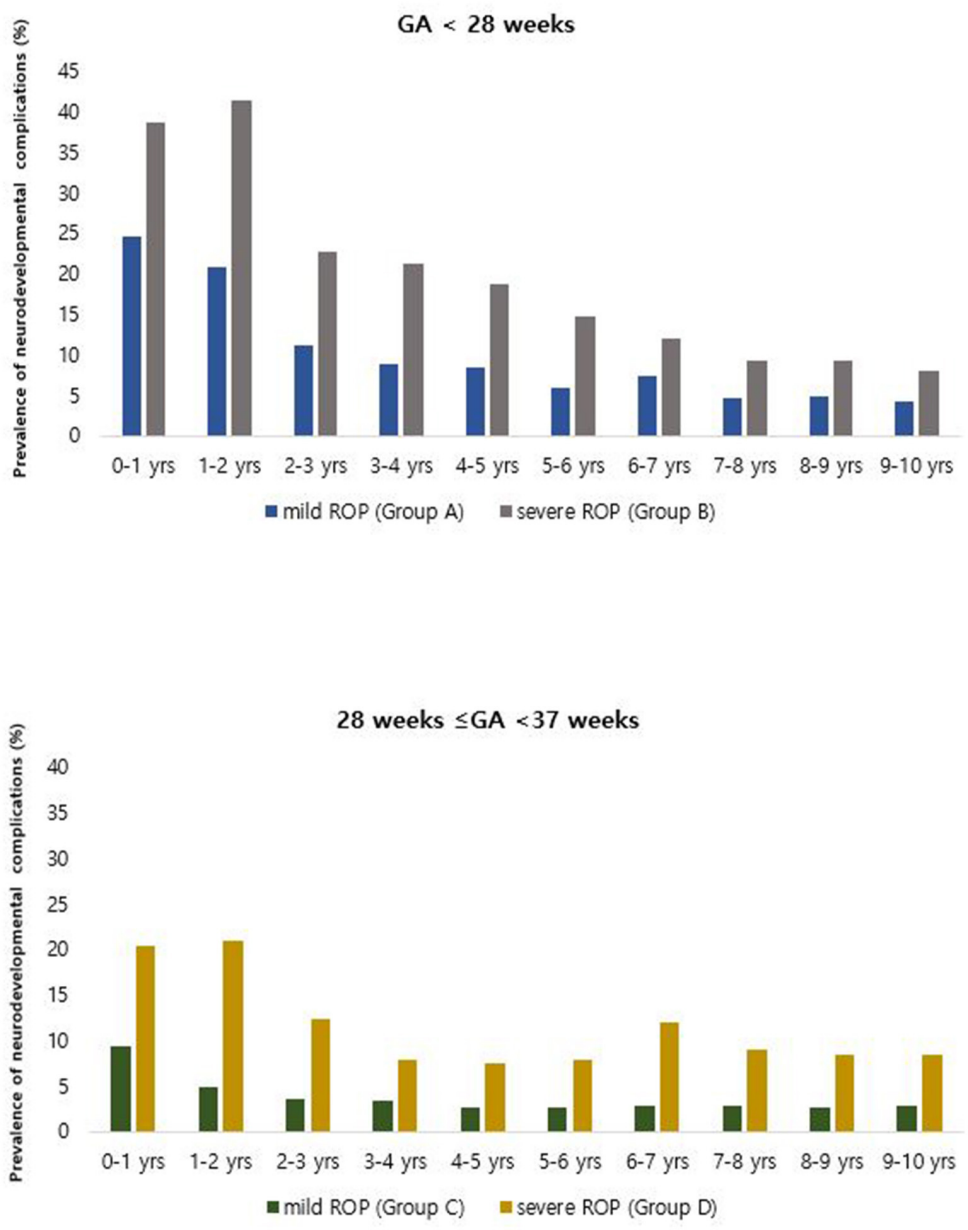
Annual prevalence of overall neurodevelopmental complications among patients born between 2007 and 2008 with retinopathy of prematurity and treated, based on the Korean National Health Insurance Service database throughout the 10-year follow-up period.

Comparing the prevalence of neurodevelopmental disorders according to ROP severity revealed that the prevalence of neurodevelopmental disorders was higher in patients with severe ROP (Groups B and D; Table 1). The prevalence of neurodevelopmental disorders in the first year of life was 24.65, 38.67, 9.28, and 20.40% in Groups A, B, C, and D, respectively. The prevalence gradually decreased after birth, from 24.65% in the first year to 4.23% after 10 years in Group A, 38.67–8.00% in Group B, 9.28–2.89% in Group C, and 20.4–8.46% in Group D (Figure 2). In both the extremely premature infant group and the other premature infant group, the prevalence was higher in the severe ROP group within the first year of life, even after 10 years.

**Table 1:** Annual prevalent number of cases and prevalence of overall neurodevelopmental complications among patients born between 2007 and 2008 with retinopathy of prematurity and treated, based on the KNHIS database throughout the 10-year follow-up period.

	**GA** **< 28 weeks**				**28 weeks ≤ GA < 37 weeks**			
	**Mild ROP** **Group A (*****N*** **= 209)**		**Severe ROP** **Group B (*****N*** **= 75)**		**Mild ROP** **Group C (*****N*** **= 6,510)**		**Severe ROP** **Group D (*****N*** **= 201)**	
**Age**	**Prevalent case (***n***)**	**Period prevalence (%)**	**Prevalent case**	**Period prevalence (%)**	**Prevalent case**	**Period prevalence (%)**	**Prevalent case**	**Period prevalence (%)**
0–1	70	24.65	29	38.67	623	9.28	41	20.40
1–2	59	20.77	31	41.33	324	4.83	12	20.90
2–3	32	11.27	17	22.67	235	3.50	25	12.44
3–4	25	8.80	16	21.33	227	3.38	16	7.96
4–5	24	8.45	14	18.67	179	2.67	15	7.46
5–6	17	5.99	11	14.67	176	2.62	16	7.96
6–7	21	7.39	9	12.00	193	2.88	24	11.94
7–8	13	4.58	7	9.33	191	2.85	18	8.96
8–9	14	4.93	7	9.33	184	2.74	17	8.46
9–10	12	4.23	6	8.00	194	2.89	17	8.46

*GA, gestational age; KNHIS, Korean National Health Insurance Service; ROP, retinopathy of prematurity*.

#### Association With Neurodevelopmental Disorders According to ROP Severity

Among extremely premature infants with ROP, those with severe ROP had a higher association with neurodevelopmental disorders than those with mild ROP (hazards ratio: 3.082; *p* < 0.001).

Among other premature infants with ROP, those with severe ROP had a higher association with neurodevelopmental disorders than those with mild ROP (hazards ratio: 3.269; *p* < 0.001) (Table 2).

**Table 2:** Crude and adjusted hazard ratios of neurodevelopmental complications according to retinopathy of prematurity severity, sex, and income level during the 10-year follow-up of premature infants.

	**GA < 28 weeks**							**28 weeks ≤GA < 37 weeks**						
	**Crude**			**Adjusted**				**Crude**			**Adjusted**			
	**HR**	**95% CI**		**HR**	**95% CI**		***P*** **-value**	**HR**	**95% CI**		**HR**	**95% CI**		***P*** **-value**
ROP	2.844	1.974	4.099	**3.082**	**2.128**	**4.465**	**<0.001**	3.16	2.561	3.912	**3.269**	**2.644**	**4.043**	**<0.001**
severity														
Sex	0.718	0.499	1.034	0.695	0.479	1.009	0.056	0.744	0.663	0.835	0.738	0.658	0.828	0.001
Income level	0.943	0.582	1.53	0.925	0.565	1.515	0.756	0.946	0.8	1.119	0.936	0.792	1.108	0.4433
Region	0.771	0.539	1.104	0.68	0.47	0.984	0.113	1.039	0.928	1.162	1.067	0.952	1.195	0.2647
Year of diagnosis	0.94	0.657	1.345	0.905	0.625	1.31	0.5966	1.074	0.96	11.203	1.099	0.982	1.231	0.1005

*The bold letters used to emphasize that the severity of ROP significantly increased the HR of neurodevelopmental complications in both GA < 28 and GA 28–37 groups. GA, gestational age; HR, hazard ratio*.

#### Detailed Neurodevelopmental Disorders in Premature Infants With ROP

We classified premature infants with ROP according to detailed neurodevelopmental disorders, including neurodevelopmental delay, neurocognitive functional, speech and language, and motor developmental disorders. Severe ROP had a higher association with each of the detailed neurodevelopmental disorders than those with mild ROP (neurodevelopmental delay, HR: 4.831; developmental disorders of speech and language, HR: 2.701; *p* < 0.001).

However, we couldn't obtain meaningful results for motor developmental delay and cognitive developmental delay due to the small number of patients.

## Discussion

In the present study, we found that the prevalence of neurodevelopmental disorders was higher in premature infants with severe ROP than in those with mild ROP, both in the first year of life and after 10 years. We tried to evaluate whether the prevalence of neurodevelopmental disorders differed based on the severity of ROP among the patients who were less likely to have neurological disorders, namely, those that were assumed to have the same possibility of suffering neurological developmental disorders. Moreover, this was the first to investigate long-term neurodevelopmental outcome of the ROP infants using nationwide population-based database.

We compared infants with a similar GA in the current study. Children with severe ROP generally have a lower GA, which is a risk factor for developing neurodevelopmental complications; therefore, this study selected patients within the same GA range with a similar probability of developing neurodevelopmental complications, classifying them based on ROP severity. We evaluated the association between developmental disorders and ROP severity in each group with similar risk of neurodevelopmental complications.

Intraventricular hemorrhage, PVL, and birth asphyxia, etc., are important risk of neurodevelopmental complications. Their severity is based on the levels and grades. To analyze the health insurance database, we used diagnostic codes to identify patients who have disorders/diseases, and the severity of IVH, PVL, and birth asphyxia cannot be evaluated based on the diagnostic codes. Thus, we had to exclude patients who were diagnosed with other neurologic disorders, such as IVH and PVL, the severity of which would have affected the occurrence of neurodevelopmental complications.

Additionally, because there are differences in the frequency of neurodevelopmental evaluation and implementation of neurodevelopmental interventions according to the area of residence (metropolitan cities vs. others) and income level of patient's household (insurance payment classes: low vs. middle and high), these factors were adjusted. Even after adjusting for income level and area of residence, we found that a higher ROP severity was associated with a higher prevalence of neurodevelopmental disorders; therefore, severe ROP is likely to be associated with developmental and neurological disorders 10 years later in premature infants.

In our study, the prevalence of neurodevelopmental disorders was the highest during the first year of life and gradually decreased. Those with additional risk factors for early death—such as congenital anomalies and birth injuries—were excluded at the time of enrollment. So our study is based on the assumption that the missing data caused by death would have had little effect on the outcome. Therefore, the decreasing prevalence observed with increasing age was not attributed to early death. Since developmental delay requires continuous observation of the child's condition and recognition of associated abnormalities, diagnosis takes a considerably long time. Additionally, some developmental disorders can only be diagnosed after a certain period of time; therefore, some disorders may not have been diagnosed during the follow-up period of 10 years (study duration). As a result, the number of diagnosed patients may be lower than the actual prevalence. Further, we have used diagnosis codes to evaluate the prevalence each year. The neurodevelopment complications included all the severity stages from the lowest to the highest and developmental delay in the early assessment may naturally improve in cases with mild developmental delay while growing up. And another possible reason is that although many patients are diagnosed with neurodevelopmental disorders at an early age, their developmental status may have improved through early diagnosis, intervention, and developmental therapy. This demonstrates the importance of early detection and intervention in neurodevelopmental disorders.

Neurodevelopmental disorders are common, with a high prevalence of about 5–10%, in children (30, 31). With the recent increase in the survival rate of high-risk infants, the prevalence of developmental disorders has also increased. As of 2016, the prevalence of developmental disabilities in premature infants was 23% (7). In the mild ROP group in our study, the prevalence of developmental delay was approximately 10%, similar to the prevalence in the general population (5–10%) and slightly lower than in the general premature infant population (23%), due to the exclusion criteria. Meanwhile, the prevalence of developmental delay in the severe ROP group in our study was approximately 25%, which is higher than in both the general (5–10%) and general premature infant (23%) populations. These findings suggest that the severity of ROP may be associated with developmental delay.

Retinopathy of prematurity is known to cause severe ophthalmic complications, such as blindness, myopia, anisometropia, amblyopia, astigmatism strabismus, and secondary glaucoma (32–35). Several recent studies have reported a correlation between severe ROP and extraocular complications, particularly neurodevelopmental complications (16, 17). Drost et al. reported that severe ROP (stage 3–4) was associated with lower cerebellar and brainstem volumes and poorer neurodevelopmental outcomes (36). Moreover, ROP requiring treatment was associated with lower fractional anisotropy in the posterior white matter and decreased maturation measures in the optic radiation, internal capsule, and external capsule (16, 18). This suggests that brain abnormalities are frequent among children with ROP (18); furthermore, ROP requiring treatment increased the risk of motor impairment, cognitive impairment, or hearing loss (16, 17). While previous studies investigated the developmental and maturity status of the brain through imaging screening at a point in time, this study additionally investigated the annual change in prevalence for 10 years using the KNHIS database, which is another strength of this study. Children grow and develop as a result of interactions between innate attributes—such as physical conditions, disposition, and cognitive abilities—and external factors, such as nutrition and environment (19, 31, 37). The molecular etiology of the relationship between the development of ROP and oxygen therapy thus demonstrates the role of VEGF in the development of retinal blood vessels, proliferation of endothelial cells, and formation and movement of blood vessels (10, 13, 38). Angiogenesis normally occurs in the physiological hypoxic environment of the uterus; however, during the administration of high oxygen pressure for therapeutic purposes after birth, transcription decreases, inhibiting angiopoiesis (14, 34, 38). By contrast, excessive transcription is induced when the oxygen pressure is low, and angiogenesis occurs in the avascular region (10, 15, 34). Whether these changes in the mechanisms of angiogenesis affect the brain or the change in visual stimuli caused by ROP affects developmental delay remain unidentified; thus, further studies with larger cohorts are required.

This was a large-scale data analysis with a sample size of 6,995 patients using data from the KNHIS database. To the best of our knowledge, this is the first study to demonstrate a relationship between ROP and neurodevelopmental disorders using a nationwide population-based database. The KNHIS database, organized by the National Health Insurance of Korea, includes all citizens of South Korea and includes data regarding their diagnoses, procedures, prescription records, medical treatment records, sociodemographic characteristics, and direct medical costs (21). It is noteworthy that all premature infants born in Korea and diagnosed with ROP between 2007 and 2008 were included in the study. Additionally, our study was designed to identify cases in which neurodevelopmental disorder was diagnosed later among patients diagnosed with ROP; therefore, we also demonstrated that ROP could be a risk factor for neurodevelopmental disorders. To prove this, future studies should confirm more precise causality. If severe ROP is determined as a risk factor for developmental delay, it is imperative to include severe ROP in the criteria of neurodevelopmental screening tests for premature infants. Classification of patients with severe ROP as a high-risk group for neurodevelopmental disorders will therefore facilitate early diagnosis and screening tests for appropriate intervention and treatment. Pediatricians currently pay great attention to use of oxygen therapy in NICU to prevent ROP (38). Additional efforts will be required to prevent possible long-term neurodevelopmental disorders.

The present study had several limitations; first, this was not a prospective cohort study. We used data from the KNHIS database, identified by diagnostic codes and registered by clinicians; the accuracy of the data may therefore differ depending on the accuracy of the diagnostic code determined by the physician and whether the code was deleted or added according to the condition evaluated yearly. Additionally, since patients with diagnostic codes indicating trauma or birth injury were excluded only in the early stage of birth, the possibility of neurodevelopmental complications due to other diseases or trauma at a later time cannot be excluded. Moreover, there could be a detection bias in the young age group during follow-up. Some neurodevelopmental complications are more likely to be diagnosed when a child can undergo a developmental test or around school-going age; therefore, prevalence—especially in younger children—should be interpreted in the context of detection bias. And it is difficult to know if a decrease in the cases with diagnosis codes indicates the actual improvement over time or faults in the early assessment. Finally, neurodevelopment complications may be related to the adverse events during the NICU stay (i.e., mechanical ventilation, NEC, PVL, IVH, etc.), which affect the general well-being of the infant. Although we set exclusion criteria to exclude infants diagnosed with other neurologic disorders which could confound the results of the present study, such adverse events could not be excluded, as it will lead to much loss of the enrolled patients. It should be considered as one of the limitations of a big data-based study based on the diagnostic codes.

Despite these limitations, we enrolled patients with the same probability of developing neurodevelopmental complications as possible. When comparing the prevalence of neurodevelopmental complications according to the severity of ROP in the group considered to have the same risk of neurodevelopmental complication, it can be regarded as reliable that the prevalence of neurodevelopmental complications was higher in the severe ROP group than in the mild ROP group.

## Conclusion

In conclusion, the present study reported on developmental delay in premature infants with ROP in South Korea using the KNHIS database, over a 10-year follow-up period. There was a significantly higher association with developmental delay in patients with severe ROP than in those with mild ROP, suggesting that severe ROP is associated with developmental and neurological disorders in premature infants. If severe ROP is determined as a risk factor for developmental delay through further research, it is imperative to include severe ROP in the criteria of developmental screening tests for premature infants.

## Data Availability Statement

The original contributions presented in the study are included in the article/supplementary material, further inquiries can be directed to the corresponding author/s.

## Ethics Statement

The studies involving human participants were reviewed and approved by Institutional Review Board (IRB no. 2018-04-001) of Hanyang University Guri Hospital, Gyeonggi-do, South Korea. Written informed consent from the participants' legal guardian/next of kin was not required to participate in this study in accordance with the national legislation and the institutional requirements.

## Author Contributions

Y-JC and EH conceptualized and designed the study, drafted the initial manuscript, and reviewed and revised the manuscript. YS, GB, and IK designed the data collection instruments, collected data, carried out the initial analyses, and reviewed and revised the manuscript. IK and HC conceptualized and designed the study, coordinated and supervised data collection, and critically reviewed the manuscript for important intellectual content. All authors approved the final manuscript as submitted and agree to be accountable for all aspects of the work.

## Funding

This work was supported by the National Research Foundation of Korea (NRF) grants funded by the Korean government (NRF-2020R1A2C1010229 to HC, NRF-2020R1F1A1048475 to YS, and NRF-2021R1I1A1A01059690 to EH).

## Conflict of Interest

The authors declare that the research was conducted in the absence of any commercial or financial relationships that could be construed as a potential conflict of interest.

## Publisher's Note

All claims expressed in this article are solely those of the authors and do not necessarily represent those of their affiliated organizations, or those of the publisher, the editors and the reviewers. Any product that may be evaluated in this article, or claim that may be made by its manufacturer, is not guaranteed or endorsed by the publisher.
